# SARS-CoV-2 neutralizing antibody development strategies

**DOI:** 10.3906/biy-2005-91

**Published:** 2020-06-21

**Authors:** Bertan Koray BALCIOĞLU, Melis DENİZCİ ÖNCÜ, Hasan Ümit ÖZTÜRK, Fatıma YÜCEL, Filiz KAYA, Müge SERHATLI, Hivda ÜLBEĞİ POLAT, Şaban TEKİN, Aylin ÖZDEMİR BAHADIR

**Affiliations:** 1 Genetic Engineering and Biotechnology Institute, Marmara Research Center, TÜBİTAK, Kocaeli Turkey; 2 Department of Basic Medical Sciences, Faculty of Medicine, University of Health Sciences, İstanbul Turkey

**Keywords:** SARS-CoV-2, COVID-19, neutralizing antibodies, human monoclonal antibody, display technologies, human scFv, neutralizing assays, animal models

## Abstract

In December 2019 a novel coronavirus was detected in Wuhan City of Hubei Province-China. Owing to a high rate of transmission from human to human, the new virus called SARS-CoV-2 differed from others by its unexpectedly rapid spread. The World Health Organization (WHO) described the most recent coronavirus epidemic as a global pandemic in March 2020. The virus spread triggered a health crisis (the COVID-19 disease) within three months, with socioeconomic implications. No approved targeted-therapies are available for COVID-19, yet. However, it is foreseen that antibody-based treatments may provide an immediate cure for patients. Current neutralizing antibody development studies primarily target the S protein among the structural elements of SARS-CoV-2, which mediates the cell entry of the virus through the angiotensin converting enzyme 2 (ACE2) receptor of host cells. This review aims to provide some of the neutralizing antibody development strategies for SARS-CoV-2 and in vitro and in vivo neutralization assays.

## 1. Introduction

In December 2019, in Wuhan City of Hubei Province-China, a novel coronavirus (coronaviridae family) was detected. Coronaviruses are members of a wide group of viruses causing various diseases ranging from flu to more extreme diseases like severe acute respiratory syndrome (SARS) and Middle East respiratory syndrome (MERS). The new virus called SARS-CoV-2 differed from others by its unexpectedly rapid spread due to a high rate of transmission from human to human. 

There are currently no approved targeted therapies available for COVID-19. Researchers worldwide are exploring COVID-19 prevention strategies and therapeutic options, including convalescent plasma, monoclonal antibodies, vaccines, peptides, interferon, small molecule drugs, as well as exploring the repurposing of proven drugs (Li and De Clercq, 2020). Vaccination may provide a strong and sustainable protection, however, vaccine development is a long and challenging process, and vaccination is only useful in a preventive environment. On the other hand, an antibody-based therapy can provide immediate effect for patients.

Neutralizing antibodies (NAbs) target viral surface proteins for blocking the attachment of virus to host cell (Klasse, 2014). Therefore, in SARS-CoV-2 studies, amongst all structural proteins, neutralizing antibodies primarily target the S (spike) protein, which mediates entry into cells. The structural protein S is a transmembrane glycoprotein that has 2 functional subunits: the subunit S1 that is involved in cell attachment and the subunit S2 that mediates cell membrane fusion (Siu et al., 2008). S1 also breaks down into 2 domains, a receptor-binding domain (RBD) and an N-terminal domain (NTD). The S protein binds the human angiotensin converting enzyme 2 (ACE2) receptor through its S1 subunit. SARS-CoV-2 appears to be using the same receptor, ACE2, for cell entry as SARS-CoV with a 10 to 20-fold higher affinity (Wrapp et al., 2020). As shown in Table, all currently developed anti-SARS-CoVNAbs target the S protein, predominantly target the RBD, while some target regions in the S2 subunit or the S1/S2 proteolytic cleavage site. S1 RBD is the most crucial target for SARS-CoV NAbs, which may interrupt the interaction of RBD and its ACE2 receptor (Wong et al., 2004).

**Table:** Strategies for neutralizing antibody development.

Methods	Original antibody	Reformated antibody	Target region	In vitro/ in vivo model	Ref
Convalescent plazma	Human antibodies from convalescent COVID-19 patients	-	Whole virus	COVID-19 patients	Duan et al. 2020; Shen et al. 2020; Xinhua 2020
	Human antibodies from convalescent COVID-19 patients	-	Whole virus	Pseudotyped virus neutralization assay	Wu et al. 2020
Hybridoma	47D11 Mouse/Human Chimeric full antibody against SARS-CoV	Fully human antibody	SARS-CoV-2 Spike antigen S1-S2 region	Pseudotyped virus neutralization assay	Wang et al. 2020
	Full antibody from mouse hybridoma	-	SARS-CoV-2 Spike antigen RBD Domain	Pseudotyped virus neutralization assay	Xiong et al. 2020
Human hybridoma	There are no NAbs developed with this technique				
	Two monoclonal antibodies (P2C-1F11 and P2B-2F6) were selected from the B lymphocyte of convalescent COVID patients.	The genes of the selected B lymphocytes were cloned into mammalian expression system	SARS-CoV-2 Spike antigen RBD domain	Pseudotyped virus and SARS-CoV-2 virus neutralization assay	Ju et al. 2020
Phage display	Single-domain antibody from llama	Bivalent human IgG Fc-fusion protein	SARS-CoV-2 Spike antigen	Pseudotyped virus neutralization assay	Wrapp et al. 2020
	Synthetic human Fab library	CDR3 Diversification by mutations	SARS-CoV-2 Spike antigen RBD	Pseudotyped virus neutralization assay	Zeng et al. 2020
	Single-domain antibody	Grafting naive CDR regions into the framework region of an allele in human antibody heavy chain variable region	RBD domain and the S1 subunit of SARS-CoV-2	Pseudotyped virus neutralization assay	Wu et al. 2020
	Naive human scFv antibody	Human IgG1 antibody (4A3)	SARS-CoV-2 RBD	Pseudotyped virus neutralization assay	Liu et al. 2020
	Domain library	Fused with human Fc	SARS-CoV-2-RBD	Pseudotyped virus and SARS-CoV-2 virus neutralization assay	Liu et al. 2020
Mammalian display	There are no NAbs developed with this technique				

Here in this review, we discuss reverse engineering from convalescent plasma, classical hybridoma technology, human hybridoma technology, phage display technology, and mammalian cell surface display technology to develop human antibodies, humanized antibodies and human scFv and single domain antibodies against SARS-COV-2 (Figure). Later we provide brief summary of in vitro and in vivo neutralizing assays including animal models for SARS-CoV-2. 

## 2. Convalescent plasma 

Although there are many projects carried out in many laboratories around the World, currently, there are no approved drugs, vaccines, NAbs, or antiviral agents targeting coronavirus, and they may not be available in a short time (Duan et al., 2020). On the other hand, the rising number of patients recovering from COVID-19 with a high neutralizing antibody titer day by day highlights convalescent plasma therapy as a promising alternative for COVID-19 treatment (Chen et al., 2020).

For more than a century, convalescent plasma (CP) therapy, which is traditional passive immunotherapy, has been used to prevent and treat the outbreaks of many infectious diseases which have been reviewed previously (Casadevall and Pirofski, 2020). 

Some clinical CP therapy experiments have already been reported on SARS-CoV-2. In China, 245 COVID-19 patients received pilot convalescent plasma therapy in February, and 91 cases demonstrated improvements in terms of clinical indicators and symptoms. Duan et al. (2020) reported the results of CP transfusion on 10 severe adult cases confirmed by a real-time viral RNA test. A single dose of 200 mL CP derived from 39 recovered donors having neutralizing antibody titers above 1:640 was transfused to the patients, and their clinical symptoms such as the oxyhemoglobin saturation, the level of neutralizing antibodies, the lymphocyte counts and the decrease of C-reactive were significantly improved. 

Shen et al. (2020) reported another CP transfusion study on 5 severe COVID-19 patients who were receiving mechanical ventilation during treatment, and all of whom had received antiviral agents and methylprednisolone. Following the transfusion of CP with a neutralizing titer more than 1:40, the body temperatures decreased within 3 days. Within 12 days, the SOFA scores were reduced, the PAO2/FIO2 ratios were increased, and the viral loads became negative. Neutralizing antibody titers of all 5 patients increased by scores ranging from 80 to 320. 

For CP therapy evaluation, the level (titers) of neutralizing antibodies against SARS-CoV-2 virus is critical, and the plasmas containing NAbs must be obtained from recovered patients when the titers of NAbs reach their peak after the onset of disease. Wu et al. (2020) screened plasma samples collected from 175 COVID-19 recovered patients using pseudotyped lentiviral vector based neutralization assay. The domain RBD, the subunit S1, and the subunit S2 of SARS-CoV-2 were used in ELISA to determine the level and time-course of spike-binding antibodies in plasma. They revealed that SARS-CoV-2 specific antibodies were detected from the day-10-15 after the onset of disease and remained thereabouts afterward. The titers of patients varied depending on age. Elderly and middle-aged patients had significantly higher plasma-NAb rates (P < 0.0001) and spike-binding antibodies (P = 0.0003) than young patients.

There are also some known and theoretical risks of passive administration of convalescent sera. Transfusion transmitted infections (TTI), allergic transfusion reactions like serum sickness; transfusion associated circulatory overload (TACO), transfusion related acute lung injury (TRALI) are the known risks of convalescent sera transmission. While, TRALI is of particular concern in severe COVID-19 given potential priming of the pulmonary endothelium the risk of TRALI is less than 1/5000 transfused units. With the development of modern blood banking methods, the risk of accidental transmission of known infectious agents or triggering transfusion reactions becomes very low (Casadevall and Pirofski, 2020). There is a theoretical risk of antibody-dependent enhancement phenomenon of infection after transfusion. ADE refers to a process in which antibodies that target one serotype of coronavirus could enhance infection to another viral serotype. Wan et al. (2020), showed a novel mechanism for ADE in which a NAb that binds coronavirus surface spike protein like a viral receptor, activates spike conformation and mediates viral entry into receptor-expressing IgG Fc cells via canonical viral receptor-dependent pathways. Also, it is still unknown to what extent convalescent plasma represses the development of a natural immune response, especially when administered for prophylaxis and makes such individuals vulnerable to reinfections. Risk-benefit assessment must be investigated carefully and must be supported with scientific data. Although CP therapy is a fast and effective form of treatment that can be applied in difficult situations, is not a real solution for diseases. Since it is associated with the amount of antibody titers in the blood of patients who recover, which is not an infinite source. Therefore, reverse engineering is needed for the development of neutralizing antibodies. 

Ju et al. (2020), are the first group which characterized 206 RBD-specific human mAbs isolated from single B cells by FACS from eight SARS-CoV-2-infected patients. They have cloned the genes of these B cell into an expression vector for further analysis. Then they showed the potential binding and neutralizing effect of the antibodies by surface plasmon resonance assay and neutralization assay with pseudovirus and the SARS-CoV-2 virus. They found 2 potent SARS-CoV-2 neutralizing human monoclonal antibodies (P2C-1F11 and P2B-2F6). 

## 3. Hybridoma technology

An ideal alternative to hyperimmune sera is monoclonal antibodies owing to their specific pharmacological and safety profiles (Traggiai et al., 2004; Li et al., 2006). Hybridoma technology allows the production of monoclonal antibodies against specific target antigens, in large quantities in the laboratory environment. 

Classical hybridoma technology includes the vaccination of mice with specific antigens and the development of hybridoma cells by the fusion of antibody-producing B lymphocytes and immortal myeloma cells. The fusion merges the cytoplasms of the 2 beneficial somatic cells with the help of a chemical agent (polyethylene glycol) (Galfre and Milstein, 1982). Effective immunization is essential to obtain a strong and target-specific antibody response from mice. Immunization is done either by using the whole/a part of an antigen or antigenic peptides for which immunogenicity is enhanced by conjugating them to a larger and immunogenic carrier molecules such as KLH, BSA or ovalbumin (Fuentes, 2005; Ertekin, 2013). 

B cells that produce antibodies in the spleen and lymph nodes of animals, immunized with the target antigen, are fused with myeloma cells to form immortal antibody-producing cell lines. The basis of monoclonal antibody technology is to ensure the recognition of a single epitope and the production of antibodies by hybrid cell lines. Therefore, the antibody activities after fusion are detected by indirect ELISA. Selected hybridomas are subjected to 3 rounds of cloning using limited dilution method. Cross-reactions are investigated by testing the antibody activity against similar viruses, enzymes and proteins, especially blood serum proteins, to determine the specificity of the antibodies

In recent studies, chimeric antibodies, containing both human and mouse antibody sequences, have been developed by hybridoma technology to treat and prevent the new types of human coronavirus MERS-CoV and SARS-CoV-2 associated diseases (Berry et al., 2004; Widjaja et al., 2019; Wang et al., 2020). 

Wang et al., 2020, screened the supernatants of 51 SARS-S hybridomas derived from immunized transgenic H2L2 mice that encode chimeric immunoglobulins (human variable regions and rat constant regions) against SARS-CoV-2 S2-S1 region. Four of the supernatants displayed cross-reactivity with the subunits S2 and S1 of SARS-CoV-2. One of these hybridoma supernatants (47D11) revealed neutralizing activity in pseudotyped virus neutralization assay. The chimeric 47D11 was then reformatted into a fully human antibody. They hypothesized that this antibody is neutralizing both SARS-CoV and SARS-CoV-2 through a different binding region than the expected RBD-ACE2 binding. 

Xiong et al. (2020) reported that SARS-CoV-2 spike RBD domain specific antibody was generated from immunized BALB/c mouse with SARS-CoV-2 spike RBD domain protein. With this study, mAbs against SARS-COV-2 RBD were produced using hybridoma technology. 

Using neutralizing mouse antibodies developed by the hybridoma technology may cause unfortunately the production of antimouse antibodies in humans (HAMA). Therefore, an antibody humanization step is necessary to reduce HAMA responses (Kim, 2012; Safdari, 2013; Ahmadzadeh, 2014). However, with the increased number of convalescent patients, the development of human mAbs from the covalescent patients has gain interest because no HAMA response is expected and the antibody humanization step is not needed.

There are many methods available for the development of human monoclonal antibodies (mAbs), including the transformation of Epstein-Barr virus (EBV), the use of phage-display libraries, the mammalian cell systems (i.e. CHO or NS0 transfectomas) to recombinantly produce mAbs by the immunization of transgenic mice carrying human Ig genes (xenomice), the molecular techniques focused on antigen-specific B cell isolation of Ig genes and finally the hybridoma technology. Each of these methods has certain advantages and restrictions that determine their use for other purposes which is described elsewhere (Li et al., 2006; Gorny, 2012).

## 4. Human hybridoma technology

The human hybridoma technology is a vital tool for production of human monoclonal antibodies. Early on, the possibility of using human mAbs for the prevention or treatment of human diseases was evident and was the driving force behind intensive effort to establish methods for human hybridoma. For this purpose, a selection of techniques could be used with varying efficiencies (Scott and Crowe, 2015).

One major obstacle to the development of human hybridomas over the years was the difficulty in collecting antigen-specific B cells to extend target populations. Antigen-specific memory B cells normally circulate in the peripheral blood at low concentrations, usually within a range centering about 1, or less, in 10,000 B cells. Therefore, it was difficult to generate human hybridoma cells, which would secrete desirable human mAbs. Recent technological developments in increasing the starting number of human antigen-specific B cells, improving the fusion efficiency and isolating new myeloma partners, and new cell cloning methods have allowed the development of protocols that make it possible to isolate B cells from blood samples and develop mAbs (Scott and Crowe, 2015). Despite technical improvements in human hybridoma technology, there are no available human hybridoma cell lines developed against SARS-CoV-2. 

The selection of a proper blood donor is a key to this process. First, there may be some ethical challenges and restrictions concerning the collection of B cells from individuals. Special precautions must be taken about the origins of B cells to ensure patients’ anonymity and privacy (Scott and Crowe, 2015). Then, a high titer of serum antibodies does not guarantee a high number of relevant peripheral B cells, but indicates a higher chance of effective mAb development (Gorny, 2012).

The use of unstimulated human peripheral blood lymphocytes (B lymphocytes) in mAb production is quite rare. On the other hand, human myeloma and lymphoblastoid cell lines could allow mAb production by means of fusion. Now we know how to increase the efficiency of fusion and consequently the efficiency of mAb production significantly by means of B-cell stimulation.

Today, there is no longer any technical limitation to making human mAbs in the broadest sense. Biological problems involving the determination of the type and nature of any synthetic or natural antibody, the advantages of different immunological compartments of B cells, and various assays for the qualification and quantification of mAbs have been extensively solved (Huang et al. 2020).

The most critical and challenging step in manufacturing human hybridomas is the fusion of desired lymphocyte populations with a myeloma partner effectively. There are 3 basic techniques used in mAb production to generate hybridomas: the use of (i) chemical agents such as PEG, (ii) fusogenic viruses, and (iii) electrical cytofusion. The most popular method used to generate hybridomas takes advantage of PEG’s fusogenic properties, while electric cytofusion is the most effective method of generating cell fusion and hybridoma (Wilson and Andrews, 2012; Scott and Crowe, 2015). There are several myeloma cell lines that are suitable for fusion with human B cells and are available in the American Type Culture Collection (ATCC). Two of those cell lines, SHM-D3327 and HMMA 2.5, are frequently used for development of human mAbs (Gorny, 2012).

The final step in human hybridoma generation is the isolation of successful fusion products in the form of single-cell clones, which is a method also referred to as biological cloning. (Scott and Crowe, 2015). There are now many approaches that can be used for biological cloning of human hybridomas. Traditionally, this was done by limiting dilution plating. More recently, advances in automated single-cell flow cytometric sorting, with indexing capabilities, have allowed fast, accurate and versatile single-cell plating. Finally, semisolid medium preparations can be used to grow single hybridoma cells as isolated, suspended colonies. This process can be highly automated with special clone picking devices and can also be carried out in an antigen-specific and semiquantitative fashion for the selection and biological cloning of high-producing human hybridomas (Scott and Crowe, 2015).

## 5. Phage display technology

Phage display technology was discovered in 1985 by the 2018 Nobel prize laureate George P. Smith. He successfully integrated a foreign DNA into a filamentous M13 phage genome such that he could display the gene product on the surface of the phage (Smith, 1985). Today, M13 phage has been widely used in phage display technology applications because of its ease of use in the laboratory. It is a virus infecting specifically E. coli bacteria. M13 phage also differs from other phages by its unusual mechanism of producing progeny by continually releasing new phages from the bacterium without killing it (Nancy and Janine, 2004; Ledsgaard et al., 2018). These 2 advantages made the M13 phage based display technology step forward amongst other phage display technologies.

The phage display technology is based on the integration of a gene encoding a peptide or a protein fused with the phage coat proteins. The most extensively used coat proteins for display are the PVIII and PIII proteins; however, other coat proteins have also been used for display (Smith and Petrenko, 1997). Because of its high copy number (-2700 copies), the PVIII protein has been only used for the display of small peptides due to conformational problems hampering capsid formation (Iannolo et al., 1995). The PIII system, on the other hand, with its low copy number (5 copies), allows the display of larger molecules such as recombinant antibodies. The first phage display system displaying antibodies was described by McCafferty et al. (1990). They successfully displayed antibody variable regions on phages by using immunoglobulin variable genes of hybridomas and B cells. Since then, phage display technology has been extensively used for the discovery of antibodies or peptides against a large variety of antigens in many fields of application such as toxiconology (Ledsgaard et al., 2018), drug discovery (Erdag et al., 2007; Mimmi et al., 2019), immunization (Bahadır et al., 2011), epitope mapping (Folgori et al., 1994) and virus or toxin neutralization (Lim et al., 2019) by using phage peptide and antibody libraries.

Antibody libraries have been displayed on phages in very different antibody formats. The main ones consist of Fab domain of antibodies, single chain Fv (scFv) which is the linear form of the variable domain of heavy and light chains, and single domain antibodies (nanobodies). The latter includes camelid VH domain and shark vNAR (new antigen receptor) domain (Cheong et al., 2020). There are 4 types of antibody phage libraries, naive library, semisynthetic, synthetic and immune libraries (Carmen and Jermutus, 2002). Naive libraries are generated from the natural antibody repertoire of donors or nonimmunized animals. Semisynthetic and synthetic libraries are generated from low diversity natural antibody repertoires by increasing the diversity with mutations in the CDR (complementarity determining region). Lastly, the immune library is generated from the antibody repertoire of immunized animals (Erdag et al., 2011) or diseased or vaccinated humans (Omar and Lim, 2018). The selection of a target specific antibody from the phage libraries is made with a method called biopanning or affinity selection. The method consists of exposing the antibody phage library to the target antigen which is immobilized on a solid surface. The phages displaying antibodies specific to the target, bind to the antigen and the nonbinders are washed away from the media. Then the target specific phages are recovered by elution for a phage amplification step by infecting fresh bacterial cells. The phages are collected for a second round of biopanning. Generally, 3–4 biopanning cycles are sufficient to select antigen-binding antibodies (Smith and Petrenko, 1997).

Since the COVID-19 pandemic emerged in December 2019, phage display technology has been intensively used for the development of neutralizing antibodies. Many different antibody libraries of different formats and strategies have been screened against SARS-CoV-2 spike protein and its receptor binding domain (RBD). Some of the studies have focused on screening previously developed libraries against SARS-CoV and MERS-CoV and finding cross-reactive antibodies. Others have performed screenings against semisynthetic or synthetic antibody libraries. Wrapp et al. (2020) reported that a previously developed a phage displayed single-domain antibody from llama, neutralizing the S antigen of SARS-CoV was also neutralizing the S antigen of the pseudotyped virus SARS-CoV-2 as a bivalent human IgG Fc-fusion protein. Zeng et al. 2020, constructed a phage displayed synthetic human Fab library (with an estimated size of 1 × 1012). The library was screened against the RBD domain of the SARS-CoV-2 spike antigen. They selected 2 antibodies with high affinity to RBD, however, only 1 showed neutralizing effect in competitive/blocking ELISA and pseudotyped virus neutralization assay. Wu et al. (2020) developed a phage-displayed single-domain antibody library by grafting naive CDR regions into the framework region of an allele in the human antibody heavy chain variable region. They made affinity selection against the RBD domain and the S1 subunit of SARS-CoV-2 and chose several neutralizing antibodies, including a “cryptic” epitope located in the spike’s trimeric interface. Liu et al. 2020, performed site-directed screening in a naive human scFv antibody library and domain antibody library by phage display against SARS-CoV-2 RBD. After several rounds of screening, they obtained 9 enriched clones from the domain antibody library and a single clone from the scFv antibody library. The scFv clone was reformatted into a human IgG1 antibody, while the domain antibody clones were fused with human Fc tag. By this way, Liu et al. revealed a potential neutralizing effect of these recombinant antibody structures with pseudotyped virus neutralization assay.

On the other hand, with the increasing number of convalescent plasma uses from COVID-19 patients, the B lymphocytes have become readily available for the development of phage displayed human scFv and Fab antibody libraries. To this end, we have initiated the development of phage displayed human scFv libraries generated from convalescent plasmas of COVID-19 patients.

## 6. Mammalian cell display

The basis of display technologies is the use of genotype-phenotype relations. These technologies frequently use microbial systems like phage, bacteria, and yeast. In recent years, mammalian cell display technology has been introduced with some advantages such as improved efficiency in protein folding and posttranslational modifications. Compared to other display technologies, mammalian cell display technology comes to the fore by allowing the scanning of functional antibody structures and making use of FACS and in situ scanning methods. The highlight is that the library size that can be used for scanning is smaller than that of other systems (˂109) (King et al., 2004).

Ho et al. (2006), transferred single chain variable fragments (scFv) of an antibody through transient (temporary) transfection to human embryonic kidney cells and expressed them on the cell surface. ScFv DNA was collected and analyzed using FACS (Ho et al., 2006) until scFv clones with the desired specificity were identified for the selection of the original antibody to the target antigen (Akamatsu et al., 2007). In the report, they showed that all IgG antibodies could temporarily be produced on the cell surface and cells that synthesize antibodies against the target antigen could be selected through selection cycles based on FACS (Akamatsu et al., 2007). Ho and Pastan (2019), announced that scFvs could also be transferred to the mammalian cell surface for affinity maturation. In the abstract of the report, the strategy for isolating high-affinity scFv specific to the CD22 antigen was identified as “mammalian cell display”. The strategy they have formed consists of the transient expression of antibodies on 293 T (HEK-293T) human embryonic kidney cell surface allowing clone selection by flow cytometry (Pastan et al., 2009).

Zhou et al., 2010, reported the display of full size IgGs on CHO cell surface. An important feature of the developed library was that recombinant DNA transfection was performed to include a single antibody replica gene in the genome of each cell. The use of the gene integration zone allowed the comparison of clones by a signal point. Along with the FACS system, this function proved to be capable of screening clones with high affinity and a high expression rate. 

Mammalian cell screening technology can also be used for antibody-based drug development for COVID-19 treatment. For this purpose, VH and VL antibody libraries can be generated with the blood samples of COVID-19 patients. Then, the libraries generated can be screened in mammalian cells against COVID-19 antigens (Beerli et al., 2008). Up to our knowledge mammalian cell screening hasn’t been applied for the development of NAbs for SARS-Cov-2. 

## 7. In vitro bioassays 

Once NAbs are developed, it is vital to test them for their neutralizing efficiency. In other words, it is crucial to show that the virus, which has the power to create an infection, is effectively eliminated/neutralized by in vitro and in vivo systems. To this end, easy and safe screening methods –like ELISA and surface plasmon resonance (SPR)– could be helpful in decreasing the number of NAbs to be tested in neutralization assays. These screening methods would give an idea about the blocking capability of the NAbs on the viral spike protein and ACE2 interaction. Thus, the neutralization assays would be concentrated on the most potent NAbs.

### 7.1. Plaque reduction neutralization test- (PRNT) 

Plate-reduction neutralization test is used to titer neutralizing antibody for a virus. The serum or antibody to be screened is diluted and combined with a viral suspension. The antibody is incubated with the isolated virus and then transferred on host adhering cells. The surface of the cell layer is coated with a layer of agar or carboxymethyl cellulose to prevent the indiscriminate spread of the virus (Schmidt et al., 1976). The concentration of plaque-forming units can be estimated by the number of plaques (regions of infected cells) formed after a few days. Depending on the virus, the plate builder units may be determined through microscopic examination, or with specific dyes that react with fluorescent antibodies or infected cells. The concentration of serum indicates how many antibodies are detected or how effective it is in the reduction of the number of plaques by 50% compared to the serum-free virus. This calculation is defined as the value of PRNT50.

Currently, PRNT50 is considered to be the “gold standard” to detect and measure antibodies that can neutralize viruses. It has a higher sensitivity than other tests, such as hemagglutination and many commercial Enzyme immunoassays. It is also more specific for the diagnosis of some arboviruses than other serological methods (Ratnam et al., 2009; Stephen et al., 2009).

One problem with this recently defined test is that the neutralizing ability of antibodies depends on virion maturation state and the type of cell used in the test. Therefore, if the wrong cell line is used for the analysis, the antibodies may appear to be effective in neutralization even if they are not in fact, or vice versa.

### 7.2. Virus neutralization assay

Virus neutralization assay is used in combination with an infection experiment, such as PRNT. This assay detects a neutralizing antibody that can stop virus replication. Sera or culture supernatants are diluted and mixed with the virus (the virus titer can be determined per PRNT50, etc.). The mixtures are incubated approximately for 45 minutes at room temperature, and then the mixture is added on suitable cell lines (Vero, Vero6, etc.) and the cells are incubated for 3 days at 37 °C (Traggiai et al., 2004; Xiong et al., 2020).

In a standard CPE (cytopathic effects) assay, a monolayer of cells is infected with a virus and then monitored for several days (or weeks) of morphological changes. The changes are expressed in distinct points corresponding to the sites of infection. Plaque assay has been the method of choice for several decades, where, shortly after the infection, the monolayer of cells is overlaid with semisolid material such as agarose. There are some variations in the plaque assays (with or without staining cells, for example), however, all of them are labour-intensive, human dependent and hands-on jobs; thus, the determination of CPE is difficult. Therefore, it would be helpful to opt for using a more efficient and higher throughput alternative to traditional CPE assays: a real-time cell analysis (RTCA) system which is based on electric cell-substrate impedance sensing technology that can monitor cytopathic effects.

## 8. In vivo neutralization assays

In vaccine and drug research, preclinical studies must be conducted on animals. The efficacy of a candidate vaccine or drug is investigated first on mice, and then gradually on larger animals. The most critical stage on the way to a product is in vivo challenge experiments. To this end, the candidate molecule is given to mice with target microorganism and its effectiveness is tested. During the global fight with SARS-CoV-2, vaccine and drug development studies have gained a momentum. However, there are not much in vivo results for this virus in the literature yet. On the other hand, model animal studies were conducted on MERS-CoV and SARS-CoV diseases that caused infections in the past years. Therefore, in vivo studies of these viruses from the same family as SARS-CoV-2 will constitute a reference for new studies.

### 8.1. Mouse model

Unfortunately, common small laboratory animals such as mice, ferrets, guinea pigs and hamsters were found not susceptible to MERS-CoV infection; because MERS-CoV receptor dipeptidyl peptidase-4 (DPP4) is a multifunctional transmembrane endopeptidase that breaks down insulin and other peptide hormones. For this reason, hDPP4 transgenic mouse model was created with C57BL/6 mice for MERS-CoV infection (Luke et al., 2016; Zhao et al., 2018; Li and McCray, 2020).

Studies revealed that several inbred species of the mouse (BALB / C, C57BL/6, B6, 129S) promote replication of SARS-CoV, creating clinical symptoms of pneumonitis (129S) and SARS (aged BALB/C) (Glass et al., 2004; Hogan et al., 2004; Subbarao et al., 2004; Roberts et al., 2005a).

In mice immunized with SARS-CoV, the peaks of the virus titer in lungs occur at days 2–3 postinjection. However, at days 5–7 postinjection, the virus is cleared in most mice (Roberts et al., 2008). In young mice, replication of SARS-CoV is not specifically associated with clinical symptoms and pathology of the disease. However, 129S6 mice were found to be more susceptible to SARS-CoV infection than BALB/C or B6 mice, with weight loss and early onset of pneumonia (Hogan et al., 2004; Roberts et al., 2008).

In BALB/C mice, old ones experience a more severe disease, which results in increased mortality, than young ones, just like the case in SARS-CoV infection in humans (Roberts et al., 2005a). Therefore, being susceptible to age-related factors in diseases, aged BALB/C mice might also be an ideal animal model for SARS-CoV studies. The clinical symptoms of BALB/C mice include weight loss, dehydration, fur structure deterioration and histopathological damages such as viral replication in respiratory tissues and pneumonia (Roberts et al., 2005a; Roberts et al., 2008).

Roberts et al. (2008) investigated the susceptibility of various types of aged mice to SARS-CoV infection. They reported significant weight losses by aged B6, 129S6 (12–14 months) and BALB/C mice at days 3–5 postinjection being immunized with 105 TCID50 SARS-CoV (Urbani strain) (Roberts et al., 2005a). The same study also revealed similar results in terms of viral replication levels and kinetics in the lungs of aged B6 and BALB/C mice. At days 5 and 6 postinjection, B6 mice infected with SARS-CoV were observed to have 10–70 times lower virus titers than BALB/C mice (Roberts et al., 2008).

The referred publications indicate that the mouse models for SARS with these animals, except for aged or immunocompromised ones, did not develop significant clinical symptoms or reasonable mortality rates. For this reason, in recent studies, a new transgenic mouse model has been introduced to mimic human disease, to conduct pathogenesis studies and to develop antiviral treatments for SARS: K18-hACE2 transgenic mice, which express human ACE2, the receptor used by SARS-CoV (McCray et al., 2007). It has been shown that the K18-hACE2 transgenic mouse model may also be useful in studies on the outbreak and pathogenesis of the disease by novel coronavirus-2019 (SARS-CoV-2). In this context, Linlin et al. (2020) have studied hACE2 transgenic and wild-type (WT) mice to investigate SARS-CoV-2 virus pathogenicity.

Challenge studies should be done carefully in animal bioSafety level-3 (ABSL-3) laboratories with personal protective equipment. To conduct a challenge study, the median lethal dose (LD50) of the infectious agent to be resisted must be known. Although Day et al. (2009) conducted studies on the median lethal dose in BALB/C mice using various SARS-CoV virus strains; regarding SARS-CoV-2, no literature is available on such studies, yet.

Therefore, as a precursor of the median lethal dose, in vitro studies should be started to determine the median tissue culture infectious dose (TCID50). Day et al. (2009) studied 3 different SARS-CoV virus strains (v2163, MA15 and Urban) with 3 different (103.5, 104.5, 105.5) median tissue culture infection doses (TCID50) in BALB/C mice. They observed the effects of these strains with different virulence factors on young and aged BALB/C through a 21-day challenge experiment. The survival rates of the animals per infection dose they received at different postinjection times and survival between the days of the defect were determined, and the virus titers in the lungs of the sacrificed animals were compared (Barnard et al., 2006; Roberts et al., 2007; Day et al., 2009). These studies revealed that the hypotheses on the median tissue culture infection doses (103.5, 104.5, 105.5) were valid for SARS-CoV (Day et al., 2009). Even if these doses are already determined, it is needed to conduct similar studies on animals to better calculate the virulence factor in terms of SARS-CoV-2.

### 8.2. Other animal models

#### 8.2.1. Golden Syrian hamsters

The golden Syrian hamster is an excellent model for SARS-CoV infection because viral replication is accompanied by pathological changes in the lungs including pneumonitis (Roberts A et al., 2005b). Following intranasal inoculation with 103 TCID50 SARS-CoV, hamsters support viral replication in the nasal turbinates and lungs. Viremia is detected at days 2-3 post-injection, and the virus can be recovered from the spleen and liver (Roberts et al., 2008). Although the disease produces histopathological symptoms in hamsters, clinical symptoms are not evident. The most striking example of this is reduced night movements in 5-10-week-old hamsters infected with SARS-CoV.

#### 8.2.2. Ferrets

Another disease that causes lung infection is influenza (H1N1). The model animal used in the vaccine studies of this disease is ferret. Studies have been conducted with these animals for SARS-CoV infection, which has the same target organ destruction.

It has been demonstrated that Ferrets support SARS-CoV replication and develop multifocal pulmonary lesions that contain 5%–10% of the lungs’ surface area (Ter Meulen et al., 2004). It was determined that populations immunized with 103 or 104 TCID50 SARS-CoV caused virus replication in the lungs at 106 TCID50/mL on the fourth day (Martina et al., 2003; Ter Meulen et al., 2004). Compared to other vaccine studies for SARS-CoV, this model revealed poor efficacy. Therefore, more studies were needed to understand its immunogenicity (Bisht et al., 2004; Chen et al., 2005). 

#### 8.2.3. Nonhuman primates (NHP)

Nonhuman primates (NHPs) support viral replication and pneumonitis with variable clinical symptoms and pathology, depending upon the species. Various strains of SARS-CoV were tested on rhesus macaques (McAuliffe et al., 2004; Rowe et al., 2004; Qin et al., 2005), cynomolgus macaques (Kuiken et al., 2003; Lawler et al., 2006), common marmosets (Greenough et al., 2005), African green monkeys (McAuliffe et al., 2004), squirrel monkeys and mustached tamarins. Studies revealed that squirrel monkeys and mustached tamarins are not susceptible to SARS-CoV Urbani infection. Among nonhuman primate models, rhesus and cynomolgus macaques were found to be the best NHPs in SARS-CoV studies.

Sinovac Biotech Ltd. (Beijing, China), a biotechnology company, has conducted a study on the protection of macaque monkeys from infection by SARS-CoV-2 by a vaccine candidate and announced that they attained a positive result. It is sure that the number of NHP studies will increase in vaccine development studies against the novel coronavirus-2019 (SARS-CoV-2) pandemic.

## 9. Conclusion

Since the first detection of the novel coronavirus-2019 (SARS-CoV-2) in the Wuhan City of China in December 2019, researchers from all over the world are investigating and collaborating for the determination of the virus structure and its mode of infection in order to find a treatment against COVID-19 and a vaccine for a longer protection. The experience from previous virus epidemics indicate that cocktail of neutralizing antibodies are promising for a stronger virus neutralization. Therefore, it is important to discover several antibodies able to block the virus, and this variety can be achieved by the use of various antibody development technologies.

Cocktail of NAbs has displayed a stronger neutralization than alone in treating both Ebola and SARS viruses. The generation of NAbs targeting various epitopes on SARS-CoV-2 would indeed be very important. In this way, combining a number of potent NAbs might reduce the possibility of the emergence of mutant strains.

## Acknowledgement

This work was initiated with the support of the Scientific and Technological Research Council of Turkey (TÜBİTAK Grant T1004-18AG020). Special thanks are extended to Melis Denizci Öncü and Filiz Kaya for their contributions to schematic representations and to Kemal Ulusoy for his proofreading.
